# Unpacking the navigation toolbox: insights from comparative cognition

**DOI:** 10.1098/rspb.2023.1304

**Published:** 2024-02-07

**Authors:** Kate J. Jeffery, Ken Cheng, Nora S. Newcombe, Verner P. Bingman, Randolf Menzel

**Affiliations:** ^1^ School of Psychology and Neuroscience, University of Glasgow, Glasgow G12 8QB, UK; ^2^ School of Natural Sciences, Macquarie University, Sydney, New South Wales 2109, Australia; ^3^ Department of Psychology, Temple University, Philadelphia, PA 19122, USA; ^4^ J.P. Scott Center for Neuroscience, Mind and Behavior, Bowling Green State University, Bowling Green, OH 43403-0001, USA; ^5^ Department of Psychology, Bowling Green State University, Bowling Green, OH 43403-0001, USA; ^6^ Institute for Biology, Neurobiology, Freie Universität Berlin, 14195 Berlin, Germany

**Keywords:** navigation, spatial cognition, cognitive map, vector, route, wayfinding

## Abstract

The study of navigation is informed by ethological data from many species, laboratory investigation at behavioural and neurobiological levels, and computational modelling. However, the data are often species-specific, making it challenging to develop general models of how biology supports behaviour. Wiener *et al*. outlined a framework for organizing the results across taxa, called the ‘navigation toolbox’ (Wiener *et al.* In *Animal thinking: contemporary issues in comparative cognition* (eds R Menzel, J Fischer), pp. 51–76). This framework proposes that spatial cognition is a hierarchical process in which sensory inputs at the lowest level are successively combined into ever-more complex representations, culminating in a metric or quasi-metric internal model of the world (cognitive map). Some animals, notably humans, also use symbolic representations to produce an external representation, such as a verbal description, signpost or map that allows communication of spatial information or instructions between individuals. Recently, new discoveries have extended our understanding of how spatial representations are constructed, highlighting that the hierarchical relationships are bidirectional, with higher levels feeding back to influence lower levels. In the light of these new developments, we revisit the navigation toolbox, elaborate it and incorporate new findings. The toolbox provides a common framework within which the results from different taxa can be described and compared, yielding a more detailed, mechanistic and generalized understanding of navigation.

## 1. Introduction

How animals navigate across complex terrain or featureless oceans or deserts, or find their way home after a foraging trip, has fascinated and puzzled people over centuries. Because successful navigation is essential to survival, understanding its mechanisms is a major goal for researchers in biology, neuroscience, psychology and robotics. Facilitating wayfinding is also the goal of human navigational tools, ranging from traditional systems using a complex mix of terrestrial and celestial cues [1] to modern global positioning systems.

The wealth of behavioural and neural data generated by these investigations, combined with formal computational specificity, offers an excellent opportunity to formulate explanations of navigation that link brain and behaviour [2]. The field of navigation, however, has been fragmented, with relatively little communication across disciplines, model organisms or levels of analysis. For example, neuroscientists recording cells may not think about, or find it difficult to tackle experimentally, the ecological validity of the behaviours they study. By contrast, zoologists seeking to understand natural behaviours in the wild may not consider the underlying neural substrates. Fortunately, this situation is changing as interdisciplinary societies and conferences arise, spawning cross-cutting collaborations. However, the variety of findings and wealth of data generated by these endeavours in many different model systems can be overwhelming, limiting our ability to derive general principles.

To cope with the vast mass of information and to facilitate synthesis across disparate fields, Wiener *et al*. [3] suggested the *navigation toolbox* as a formulation of common underlying principles that may operate across many different taxa. The toolbox delineates four hierarchically organized levels of spatial representation that enable the classification of navigational computations and behaviours, allowing cross-taxa comparisons. We believe this framework is useful for organizing the ever-expanding mass of data concerning animal navigation, allowing us to derive underlying principles as well as to resolve controversies. However, as research progresses we recognize that the original framework was overly one-directional, because emerging evidence is that the levels interact bidirectionally.

Here, we review and develop the framework, integrating more recent findings across different domains and exploring this bidirectionality. We first present a précis of the toolbox proposal, and then elaborate using case studies. Along the way, we illustrate how the framework addresses controversies and discuss top-down processes. We finish with discussion of how the framework can unify the study of navigation across taxa and across levels of description, and implications for the evolution of goal-directed wayfinding, adding some suggestions for new research directions.

## 2. The navigation toolbox

The navigation toolbox comprises a hierarchy of representations and processes that are organized according to their degree of *spatiality*: that is, the degree to which sensory information is processed to form representations encompassing the metric properties of distance and direction. We briefly describe these below before progressing to a more detailed deconstruction of each one.

**Figure 1.**
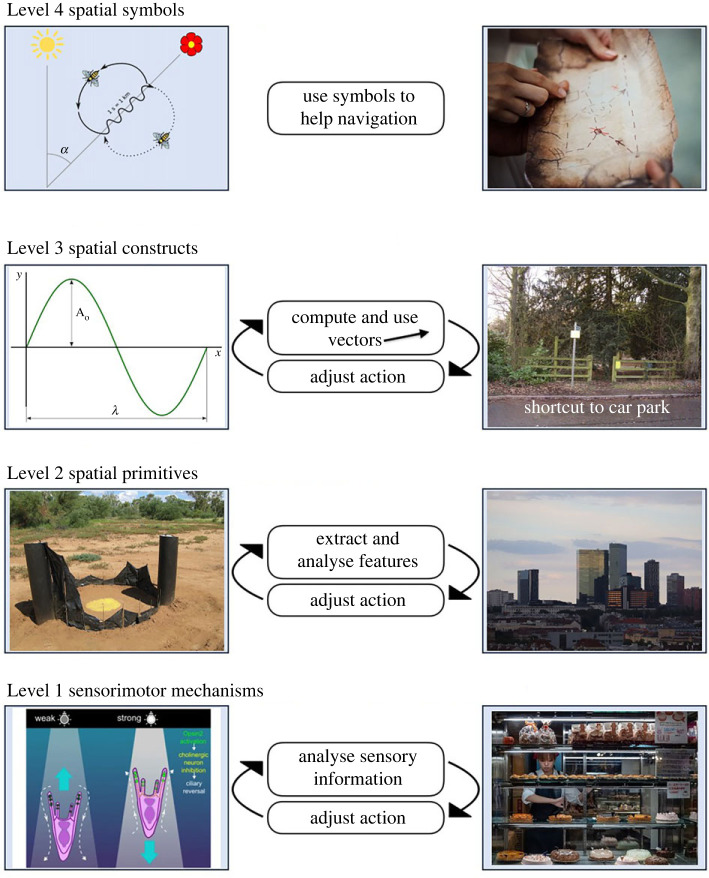
The four levels of spatial representation. The left column shows illustrative cases with non-human animals and the right with humans. At Level 1, sensory information is used directly to guide the actions used in navigation. Left: a sea urchin larva swims upwards until the light level crosses a brightness threshold, whereupon it reverses (from [4]). Right: in a strange city, humans might ‘follow their nose’ to a bakery. Level 2: simple spatial features are extracted from sensory information. Example shows the use of views to guide navigation. Left: an ant at the start of a journey home using an artificial panoramic skyline for guidance that mimics the actual skyline [5]. Right: in open space, humans might also use the broad panorama for guidance. Level 3: spatial primitives of direction and distance are combined as vectors to guide navigation. Left: *Drosophila* flies may code and transform vectors in the form of spatial sine waves [6]. Right: Humans can learn to compute shortcuts to non-distinct locations, travelling in a particular direction for an approximate distance to reach their destination. Level 4: symbols constructed or communicated by others help in navigation. Left: the renowned waggle dance of the honeybee. Right: humans often use maps to help navigation. Figure credits. Level 1, photophobia in sea urchin: from [4], the open-source publication (licence: https://creativecommons.org/licenses/by/4.0/). Level 1, bakery: from Wikimedia creative commons (licence: https://creativecommons.org/licenses/by-sa/4.0/deed.en), author: Reinhold Möller. Level 2, skyline for ants: photo by Paul Graham. Level 2, city skyline: from Wikimedia creative commons (licence: https://creativecommons.org/licenses/by/4.0/deed.en), author: Marte007. Level 3, sine wave: from Wikimedia creative commons (licence: https://creativecommons.org/licenses/by-sa/3.0/deed.en), author: badseed, using work by Josemontero9 and José Luis Gálvez. Level 3, shortcut: from Wikimedia creative commons (licence: https://creativecommons.org/licenses/by-sa/2.0/deed.en), author: Anthony Vosper. Level 4, waggle dance: from Wikimedia creative commons (licence: https://creativecommons.org/licenses/by-sa/2.5/deed.en), author not named. Level 4, treasure map: from Pixabay (licence: https://pixabay.com/service/license-summary/), author: Pexels.

The four levels (figure 1) are:

(1) sensorimotor mechanisms;
(2) spatial primitives;
(3) spatial constructs;
(4) spatial symbols.

At the *sensorimotor* level, the key process is primary sensation, sometimes directly linked to locomotor behaviour. This information is not yet processed into more elaborated constructs such as object representation, but can be used directly to guide travel, in processes such as chemotaxis (chemical guidance) [7] and phototaxis (light guidance) [4]. At the second, *spatial primitive* level, sensory information is combined into conjunctive representations that encode information having isolated spatial components—for example, distance, or direction, or landmark specificity—but lack the two- or three-dimensional aspect required for true localization.

Linkage occurs at the third, *spatial-construct* level. Here, spatial primitives are combined into representations that allow precise localization in two or three dimensions. A vector is a spatial construct: it combines distance and direction concerning how a point in space such as the current position, home base or starting point of a journey relates to another point. A route can also be a spatial construct if segments of the route comprise vectors between points. Another type of spatial construct is a cognitive map, in which a spatial location is uniquely represented in a ‘coordinate system’ (loosely speaking) such that an animal can represent, in principle, any location in the space. Finally, at the *symbolic* level, arbitrary symbols are used to help guide movement. Humans use spatial symbols all the time: words, maps or signs. Some animals may use symbols too. As a prominent example, the honeybee uses movements of its body, in the form of the waggle dance (figure 1), to ‘symbolize’ the distance and direction to a patch of flowers. However, the waggle dance is a closed system that is not capable of expanding to express new understandings or integrate spatial with non-spatial information.

We now detail, below, the characteristics of each level, illustrating each one using different taxa and different levels of analysis. We also discuss what adaptive behaviours are enabled at each level and how the levels interact with one another. We hope this framework will create a common language that enables researchers studying a wide range of navigational behaviours across many different organisms and settings to share their insights more widely.

### (a) Level 1: sensorimotor tools

The lowest level in the hierarchy, the *sensorimotor* level, relies on multimodal sensory signals. The modalities for a given species depend on the organism's suite of sensors, which furnishes its subjective sensory world (or *Umwelt*) [8,9]. The suite might include vision, olfaction, audition, touch, thermoreception, chemoreception, electroreception, magnetoreception and body senses: signals that code linear and angular acceleration, gravity, internal postural signals (proprioception) and sensations of movement (kinaesthesis).

**Figure 2.**
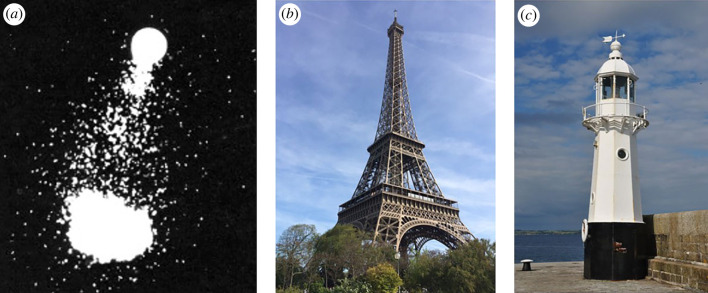
Sensorimotor processes allow simple forms of navigation towards or away from stimuli, such as in chemotaxis or beaconing. (*a*) Chemotaxis in algae in response to the pheromone lamoxirene [11]. (*b*,*c*) Beacons, positive, encouraging approach (*b*), and negative, encouraging avoidance (*c*). Figure credits: (*b*) image from https://commons.wikimedia.org/wiki/File:Eiffel_Tower_-_Paris_-_2016.JPG by Brian Lee is licensed under the Creative Commons Attribution-Share Alike 4.0 International license. (*c*) Image from https://commons.wikimedia.org/wiki/File:Mevagissey_lighthouse_(9453).jpg by Nilfanion is licensed under the Creative Commons Attribution-Share Alike 4.0 International license.

These signals provide an organism with information pertaining to places, directions and/or movement through a space, but do not fully encode these things, and so are not in and of themselves spatial. These signals alone suffice, however, for the simplest kind of navigational behaviour: movements up or down a sensory gradient, a process often called orientation (by contrast with *bona fide* navigation [10]). Such mechanisms include taxes and kineses. In taxis, the orientation mechanism works to turn an organism to a different, likely better, direction of travel; in kinesis, the mechanism changes the rate of specific, targeted behaviours based on sensory-gradient information. For example, diverse animals are attracted to light (figures 1 and 2), moving towards higher light intensities in phototaxis [4]. Scent-tracking, in which an organism follows a chemical trail to a food source, mate or home, is a common Level 1 behaviour, found in many animals, including insects [12], rodents [13], birds [14] and humans [15]. Chemotaxis, in small and microbial organisms, orients such organisms up or down chemical gradients (for example fly larvae [16]).

Level 1 information can be characterized as one dimension: the level of a single parameter such as chemical concentration, light level or wind direction. Elevating sensory information into more spatial parameters such as a direction of travel or a speed of travel takes us to Level 2: spatial primitives.

### (b) Level 2: spatial primitives

Multiple kinds of one-dimensional sensory information can combine at Level 2 to form *spatial primitives*. Level 2 primitives in our scheme include such building blocks for navigation as landmark identification, scene and context recognition, direction sense, travel-distance estimation, perception of terrain slope and detection of boundaries. Some primitives are arguably more complex and ‘constructed’ than others, but are not in themselves able to provide two- or three-dimensional positional or vector information.

One illustrative example is landmark recognition, as when recognizing the Eiffel Tower while exploring Paris. The process of moving towards such a familiar landmark, or ‘beacon’, is referred to as *beaconing* and is a simple form of navigation, akin to taxis (even garnering the term *telotaxis*) except that it requires more deeply elaborated neural processing of the cue (for example, by object recognition mechanisms). A landmark can also be used as an avoidance cue (figure 2*c*).

An example of the use of spatial primitives is desert ants matching the skyline of the panoramic visual scene to a stored memory of the scene to derive the best direction of travel (figure 1; [5]). Some ants use terrestrial cues, such as visual panoramas, to orient in a chosen direction [16–18]. Panoramic cues may guide ant navigation directly, akin to route instructions: each given panorama becomes linked with an associated heading direction. The insect does not localize its current location on a map; it just computes its direction of travel [17,19–21]. This thus falls short of linking distance and direction to form vectors.

Another important spatial primitive is the celestial (sky) compass in insects, most studied in hymenopterans [22,23]. It has three main components [24]. The dominant cue is the pattern of polarized light in the sky [25], but insects also use the position of the Sun and the spectral pattern across the entire sky [24]. Brightness and spectral composition differ according to the position of the Sun.

**Figure 3.**
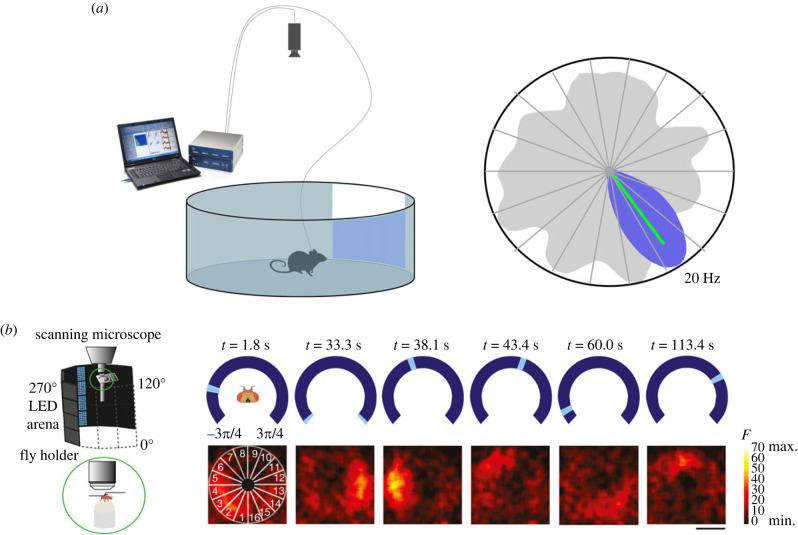
The neural basis of the spatial primitive of direction, recorded in two different taxa: rodents and insects. (*a*) Left: neurons from one of the head direction (HD) cell regions are recorded as a rat explores a cylindrical chamber in which direction is indicated by a single landmark (white card on the wall). Right: polar plot of the firing rate of a single HD neuron is plotted as a function of the facing direction of the animal. Accumulated time spent facing each of the possible directions is shown in grey. This neuron fired maximally (green line) when the rat faced ‘southeast’ (blue plot). (*b*) Neuronal activity recorded by a scanning microscope from a fruit fly as it ‘walked’ on an air-cushioned ball that controlled a video display simulating a real environment. Right (top): plots showing successive points in time as a visual cue (pale blue bar) moved around the screen, coupled to the actions of the fly. Right (bottom): heat plots showing hot-spots of neural activity in the circular region of the fly brain that maintain a consistent relationship to the visual cue. The same effect was seen in darkness (not shown), indicating that this is not just a visual response, but one that integrates the sensorimotor tools of vision and locomotion. Figure adapted from fig. 1 of [26]; published with permission (please note the rights are held by a third party).

Neurobiology has shed light on the somewhat modular nature of these abilities. Cells with direction-specific activity (figure 3) have recently been found in the ellipsoid body of the *Drosophila melanogaster* central complex [27]. This extraordinary discovery echoed the earlier discovery of ‘head direction cells' in mammals [26,28,29], which support local, short-range navigation [30–32] (and possibly long-range navigation, although this has not yet been studied). It remains to be determined whether these parallel phenomena reflect a direction sense that evolved long ago or the convergent evolution of an important spatial competence in two diverse taxa, but the similarity is striking.

Behaviourally, the compass mechanisms employed by birds are among the best-understood Level 2 directional primitives. They are reliant on celestial and/or geomagnetic cues [33]. For some nocturnal songbirds, directional compass information determines the flight paths of first-time migrants [34,35]. During migration, they fly in a specified direction for an innately programmed amount of time, a mechanism that resembles the encoding of a vector; indeed, this process is often referred to as ‘vector navigation’. The encoding of a vector makes this process Level 3, but the compass component is Level 2. In experienced migrants, the compass mechanisms are typically coupled to avian maps of space encoding distances and directions, which are more complex Level 3 spatial constructs (see below). For so-called map-and-compass navigation in birds, this Level 2–3 recursive interaction reflects a bidirectional flow of information between levels, a point we return to.

The avian hippocampus also contains head direction cells [36,37], but it is not yet established whether these support compass navigation in birds. Hippocampal lesions do not disrupt Sun-compass orientation in homing pigeons [36] nor geomagnetic compass orientation in migratory savannah sparrows (*Passerculus sandwichensis*, [36,37]), so possibly these long-range directional behaviours are supported by a different neural system in vertebrates.

**Figure 4.**
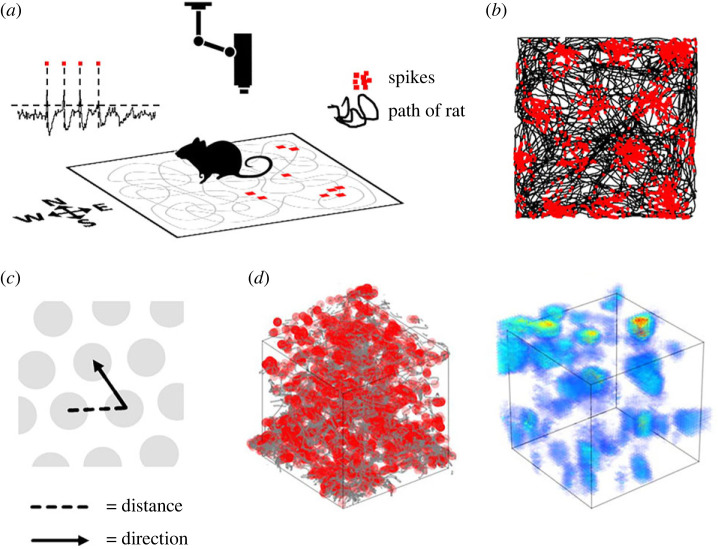
Activity of rodent grid cells. (*a*) Schematic showing the recording of a single entorhinal grid cell. A rat explores a square arena, with its path (grey line) tracked by an overhead camera. Inset oscilloscope trace shows neuronal spikes (action potentials; highlighted with red squares) from a single neuron. The red squares on the arena depict those same spikes placed at the location where the rat was when they were emitted. They congregate in restricted regions of the arena. (*b*) When the spikes plotted as in (*a*) are accumulated over a trial they form regularly spaced firing fields, indicative of odometry (distance-tracking). For simple symmetrical environments like this square platform, the firing fields form rows with a stable orientation. (*c*) The regular spacing indicates the integration of both distance and direction. (*d*) In three dimensions, this regularity breaks down, although the discreteness of the firing fields remains [41].

Another well-studied spatial primitive is travel-distance estimation (odometry). Here again, neurobiological studies have pointed to a modular separation of this function in the vertebrate brain. In mammals, grid cells in the entorhinal cortex are thought to support odometry [38–40]. Grid cells (figure 4) are neurons found in the entorhinal and parahippocampal cortex of rodents (and likely other mammals [42–44]) that increase their firing rates (production of action potentials, or ‘spikes’) when the animal enters any of multiple, evenly spaced circular regions of the environment. These spikes-in-locations are called firing fields, and together these fields often make a uniform pattern across the space (figure 4*a*), which is grid-like, hence the name. The even spacing of these fields indicates a capacity of the neurons to track distance. If animals homogeneously explore a symmetrical open field, then the firing fields align in rows with a specific orientation (figure 4*b*), which indicates integration of distance with direction (figure 4*c*) and thus amounts to a primitive form of spatial localization, approaching a Level 3 spatial construct. However, the regularity breaks down in three-dimensional space (figure 4*d*) [41,45]. The regular grids seen in restricted laboratory spaces may therefore possibly be a side-effect of the process that generates discrete fields rather than an integral part of the computation supported by grid cells (which remains unknown). Grid cells thus arguably lie on the Level 2–3 boundary.

How the distance-tracking of grid cells is achieved is not yet known, but the presence of co-localized speed-sensitive cells in this region [46] suggests a speed signal of some sort: another spatial primitive. These self-motion computations based on internally derived cues such as the vestibular acceleration sense or motor commands are sometimes called *idiothetic* [47]. They can be used directly to control movement—for example, to enable a fly to keep walking in a straight line [48]—or combined with other cues, as discussed in the next section, to enable truly spatial computations such as self-localization.

In insects, odometric information can arise from different sources: e.g. from optic flow in bees [49], or step-counting in desert ants [50,51]. Optic flow is one measure of distance travelled in flying insects like the bee: additional inputs contribute [52], such as the sequence of objects passed by, or the location of the goal in relation to other spatial features (see below). In step-counting, some parameters associated with rhythmic walking are integrated [51]. Similar parameters might account for distance-tracking in mammalian grid cells [53].

**Figure 5.**
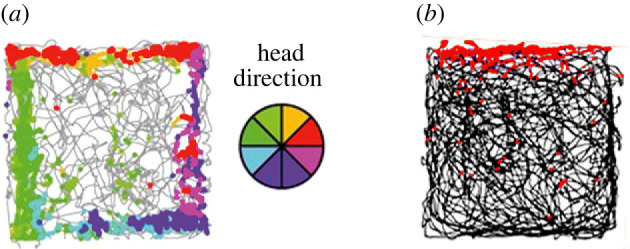
Two forms of border-related firing: egocentric (*a*) and allocentric (*b*). The plots show spikes for a single neuron overlaid on the path of a rat in grey, as previously. The egocentric boundary cell in (*a*) was recorded in the retrosplenial cortex [55]. It is ‘egocentric’ because the cell fires when the border has a given directional relationship to the animal, as shown by the colour coding. The boundary vector cell in (*b*) only fired when the rat was against a boundary lying at a given allocentric direction (e.g. north).

Another spatial primitive is boundary detection. Several neurons in the rodent spatial system appear sensitive to boundaries. For example, grid cells will stretch the distance between their firing fields if the walls of a familiar environment are moved apart [54], and place cells (discussed in the next section) also stretch their firing fields. Interestingly, the amount of stretch is only around half that of the environment, indicating a tension between the external spatial cues and the internal odometric ones. The discovery of boundary-sensitive neurons (figure 5) in several regions of the spatial system, including the subiculum [56] and entorhinal cortex [57], provides a possible mechanism for the anchoring of the spatial representation to the environment.

So far, we have presented Level 2 as deriving from Level 1 information in a bottom-up fashion. Evidence suggests, however, that besides a bottom-up flow, constructs at higher levels can also exert top-down influence on those at lower levels, mediated by bidirectional interconnections between sensory areas [58] and top-down projections from so-called ‘higher’ brain regions [59]. This bidirectional conclusion is supported by growing electrophysiological evidence of the modulation of more primary phenomena by higher-order ones. For example, experiments on mice using virtual reality showed that the responses of neurons in the primary visual area, Level 1 sensory phenomena, are modulated by running speed, a Level 2 code [60], and by spatial position, a Level 3 construct [61], with cells responding differently to the same visual stimulus depending on where the animal ‘was’ (metaphorically speaking) in global (virtual) space.

### (c) Level 3: spatial constructs

At Level 3, spatial primitives are combined to represent ‘place’. A sense of place falls in Level 3 if the place is encoded as being at certain distances and directions from other places, either precisely (metrically) or approximately (topologically). Simply recognizing a place as familiar is not enough: there needs also to be the incorporation of relational information. One way of achieving this is the equivalent of what is contained in a vector: the combining of distance and direction together. Such a representation encodes metric relations—distances and directions—between at least two locations. We consider here three such constructs: vectors, (some kinds of) routes, and maps.

#### (i) Vectors

A vector is a mathematical entity possessing a length (distance) and a direction, therefore requiring the integration of information in these two domains. Vectors provide a means to define a spatial relationship between two points in the environment.

One famous example of vectorial spatial representation is the waggle dance of the honeybee (figure 1), a spectacular Level 3 process. Nobel-Prize-winning work by von Frisch demonstrated that honeybees compute the vector between the hive and a food source [55,62–64]. These insects then communicate the vector to their hive-mates via the waggle dance. The slant of the waggle on a vertical surface indicates direction, while the duration of the waggle indicates distance. Dance-monitoring bees integrate the distance and direction components into a vector, which then guides their own behaviour, enabling them to fly in a given direction (specified by the sky compass) for a given distance (based on optic flow) to reach a food source or a new potential nest site. Note that using waggle-dance information to navigate arguably falls into Level 4, using symbolic information communicated by another animal. Recruits, however, do not only use the dance-communicated vector but also location-specific information present *en route* to the goal (see below in §2c(iii) 'Maps'). Recruited bees cope with detours [65], displacement of the starting location and changes of the Sun-compass-related reference [65,66] and they can perform shortcuts between a previously experienced location and dance-communicated locations [62].

As described earlier, the first-time migration of migratory birds is generally thought to be guided by an inherited/genetic programme, which predisposes a particular direction to fly for a defined length of time. That information is sufficient for naive migrants to approximate arrival within their population-specific overwintering range. This navigational mechanism is referred to as ‘vector navigation’ [34,63], an approximate vector that perhaps just falls into Level 3.

The ubiquitous navigational tool of path integration illustrates well the distinction between Levels 2 and 3. Homing by path integration is an example of vector navigation. Homing entails direct travel back to an invisible starting point (such as a nest) after a circuitous outwards journey, and it often makes use of path integration, which is the process of continuous updating of self-location. Path integration has been intensively studied in small mammals [64] and also insects [22], and it has been shown that the animal simultaneously knows both the direction *and* the distance back to its home: that is, it has a homing vector. Since it can execute that journey at any time (for example when suddenly startled), it must carry a continuously updated record of the homing vector.

Path integration-based homing sometimes sits between Level 2 and Level 3 processing. In homing by path integration in ants, for example, an animal follows some internal instructional motor programme to move in a particular direction for a particular distance. Is the output of such a system treated as a vector (a Level 3 construct) or is it only a single instruction to move in one direction until the strength of the signal wanes, perhaps best considered a Level 2 mechanism? Flies have now been found to transform their head direction representation [27], combined with optic flow information, into a ‘travelling direction’ [6]. This transformation of coordinate systems requires explicit representation of distance and direction, thus showing that flies represent a vector, a Level 3 construct. Path integration in rodents is also taken to explicitly encode distance and position, contributing to self-localization, and thus falls into Level 3 in our scheme.

In mammals, neurobiological work suggests that some neurons code vector signals. Boundary vector cells fire when the animal is positioned at a given vector from a boundary. They come in two forms, allocentric and egocentric (figure 5). Allocentric boundary vector cells fire when the animal is located at a given, small perpendicular distance from a particular boundary [67] and are thought to provide information that enables place cells (described below) to localize their firing [68]. Egocentric boundary cells fire when the boundary is located in a given direction with respect to the animal (left, right, straight ahead, etc.) at a given distance [69,70] and may be part of the transformation of information between the egocentric and allocentric reference frames [69–71]. Other neural vector signals include object vector cells [72,73], which fire when the animal is at a vector from a specific object, goal vector cells, which respond similarly but for goals [74,75], and ‘home base vector’ cells, in the retrosplenial cortex of mice, which fire when the animal is at a specific vector relative to its home base [76].

#### (ii) Routes

Route navigation arises when a navigator chains together a set of actions linked to environmental stimuli such as landmarks, in order to reach a goal. Route-following behaviour can comprise Level 2 or Level 3 components, or a mixture. A series of instructions linking a cue to a given travel direction would in our hierarchy be a Level 2 route. Such instructional series can form a route for ants [20–22] or sea turtles [18,77,78], with ants using panoramic views and turtles using the Earth's geomagnetic cues (inclination and intensity). We expect, however, that these route-travelling animals would quickly learn the approximate distances of each segment of their route such that the segments would then comprise vectors, at which point they are more truly spatial (Level 3).

**Figure 6.**
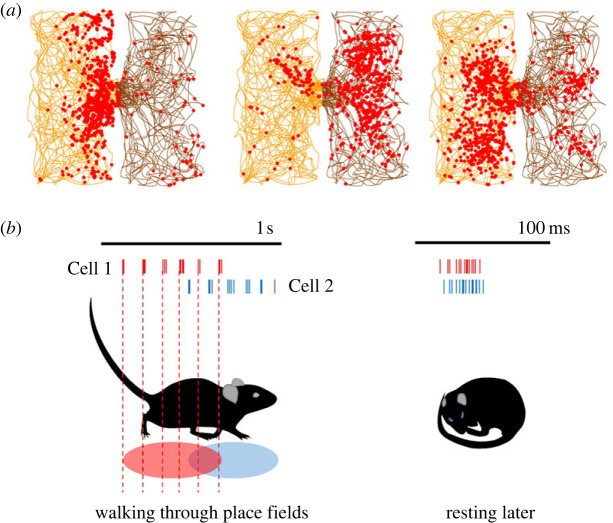
Place cells. (*a*) Data from a rat exploring two connected boxes as shown in figure 4 but with the path of the rat in yellow for one box and brown for the other. The red dots are spikes from each of the three simultaneously recorded neurons. Note that the cells fire differently in the two boxes, and also that their firing locations overlap considerably. (*b*) Place cells also encode sequences of locations. In the schematic, a rat is either walking (left) or resting/sleeping (right). The spikes from two cells are shown, aligned along the path of the rat and also across time. Left: during walking, the spikes occur sequentially in both time and place. Right: during resting, the same temporal sequence of spikes spontaneously recurs, suggesting reactivation of ‘memory’ for the route.

The hippocampal place cells may be involved in route encoding. These cells (figure 6*a*), first discovered in rats [79], fire in focal places in the environment. Each cell is active in many spaces (compare the left versus right boxes in figure 6*a*) and each given location has many cells active there (compare the three cells in this example). A sequence of traversed places forms a route, and place cells encode routes (figure 6*b*). After an animal has traversed a sequence of places, activating place cells in a temporal sequence, these temporal sequences later recur spontaneously [80], indicating memory of the spatial sequence.

#### (iii) Maps

Loosely speaking, when a representation of locations captures some relational properties of these locations, the result is a ‘cognitive map’ [81]. The existence and nature of cognitive maps have been much debated [82] but most investigators agree that a map implies, at a minimum, having multiple locations represented on a single coordinate system such that an animal can flexibly compute navigational paths between arbitrary points in the space. There may also be intermediate representations: for example, a vector map with multiple fixed points and multiple vectors, but which does not represent the entire space in a common reference frame [64,82–84]. In migrating birds, a sense of place has been suggested for experienced migrators, in contrast to the vector navigation of first-time migrators [74,85]. Displaced experienced migrators change course to head to their usual destination, while naive birds continue in the same compass (vector) direction. Experienced birds are thought to have a map sense of where in the world they have been displaced to.

The map sense of birds has been most carefully studied in homing pigeons. What emerges from that research is that a complex mosaic of maps operates over different distances, with different properties and varying dependence on the hippocampus. Best known among homing pigeon maps is the so-called ‘navigational map’ [86,87], which enables homeward orientation from distant, unfamiliar locations and appears learned from predictable variation in the distribution of atmospheric odours [88]. The navigational map also has properties that can be captured in a simple algorithm [89] that relates the olfactory profile at the home loft with the olfactory profile at a distant location [63,88], and it is, surprisingly, *not* dependent on the hippocampus [90–92]. When navigating over familiar spaces, homing pigeons can rely on visual landmarks and landscape features experienced on previous flights. However, the implementation of modern Global Positioning System (GPS)-tracking technology has suggested that how those landscape features are represented depends on whether a pigeon has a hippocampus or not. Hippocampal-lesioned pigeons can only use a familiar landscape scene to recall a Level 2 compass direction to fly off in the home direction, in what has been called ‘site-specific compass orientation’ [93]. By contrast, pigeons with a hippocampus can use the landscape scene directly to navigate a route home, using known landmarks or landscape features. This type of navigation has been referred to as *pilotage*. Hippocampal-dependent pilotage also enables pigeons to re-orient when needed [94]. Both the odour-gradient map and pilotage can be characterized as Level 3 spatial constructs; they provide positional information and have map-like properties. However, only the corrective re-orientation enabled by hippocampal-dependent pilotage has the properties of a cognitive map.

Displaced honeybees can also head towards either their hive or a feeder, after flying off a vector based on path integration [95,96]. In the most recent such detour study [96], Wang *et al*. recruited honeybees flying to a food source based on a nest-mate's waggle dance, and displaced the recruits to various locations. These displaced recruits flew first a shortened part of the vector indicated by the waggle dance and then towards the food source indicated by the dancer. Many of them crossed over this indicated location. To test whether the recruit embeds the vector information into its landscape memory, the recruited bees were moved to other places within the explored area and released [96], and their flight paths were recorded. The flight component controlled by the learned vector changed according to the difference between the expected area when starting at the ive and the experienced area, and the search flights were directed towards the location defined by the endpoint of the danced vector. Thus, the communication process enables recruits to approach the indicated location from different locations within their familiar territory. To the authors, this ability implies knowledge of not only a vector to fly, but also a location in physical space associated with the vector based on map-like information about their surroundings.

In mammals, the discovery of place cells was taken as strong evidence for a hippocampally localized cognitive map [97]. The distribution of place fields covers the entire space, but the ‘map’ (pattern of place fields produced by the population of cells active in that environment) differs between environments, meaning that the code is a population code: multiple cells are required to decode location.

Humans may map spatial relations among places using a common framework (i.e. by forming a cognitive map) although whether or not they do so in a given situation likely depends on the environment as well as the individual's abilities, motivation and experience [84]. An alternative model for how they represent spatial relations is the *cognitive graph*, in which links between key nodes are encoded along with some local metric relationships [83,98], preserving topological relationships but without the completely metric framework. Global and consistent metric relations between places on such a cognitive graph may not be found. Cognitive maps and cognitive graphs may be supported by different systems in the brain [92].

As with Level 2, interactions between levels are top-down as well as bottom-up. A top-down example in Level 3 concerns the interaction between the hippocampal spatial-context signal (Level 3) and the head direction cells (Level 2). Although head direction cells are needed for place cells to form their maps, this map can in turn drive HD cells [86,99].

### (d) Level 4: spatial symbols

We can add one more layer to the toolbox with the ability of some species, most notably humans, to make and use external symbolic devices and systems to navigate, i.e. spatial symbols (Level 4). Symbols include physical maps, signage (such as location markers, exit markers or signposts on a walking trail) and language, which can describe relationships between places and landmarks and formulate route instructions. Some of the linguistic instructions now come not from other humans, but from computerized navigational systems: for instance, SatNavs directing drivers to turn left in 100 m. The invention of tools to guide navigation has a long history, both as maps and mapping conventions became progressively more refined [100], and also as tools for measuring constructs such as time and longitude were invented [101].

The question of how human language, at Level 4, affects spatial cognition (and human thought in general) has generated long and continuing debates. Languages vary in whether they encode spatial relations in allocentric terms (north, south, east, west, etc.) or egocentric terms (left, right) or both. Some investigators claim that linguistic systems at Level 4 constrain access to Level 3 representations [102]; others suggest that the language one speaks merely biases the initial construal of what one hears, along with possibly biasing attention at encoding [103,104]. Similarly, in cognitive development, some claim that learning language is unique to humans in spatial re-orientation [105]; others suggest that language is only one part of development, which also leverages the power of human capacities for adaptive cue combination [106].

The question arises as to whether species other than humans use symbols. Arguably, the honeybee navigation system exploits symbolic communication in the form of the waggle dance, which communicates an outbound vector originating at the hive. We consider the waggle dance to contain symbolic information; this symbolic information is read by the recruit that has explored the environment and established a map-like representation of space around the hive (see above). The waggle dance is thus like human language in that it can transmit information from one brain to another, although the former is genetically encoded, whereas the latter is culturally constructed: the *capacity* for language is genetically encoded but the actual language is culturally developed and transmitted.

The symbolic devices described above differ from each other in fundamental ways. Maps provide a simultaneous overview of multiple spatial relations and supply continuous metric information. By contrast, language is inherently sequential (we can only say one thing at a time) and hence places a burden on working memory. In addition, language is often categorical, so that two locations are ‘close’ or ‘far’ from each other, with an unspecified and possibly shifting metric.

Although there has been controversy as to whether Level 4, with its human-made techniques, transforms Level 3 computations, Level 4 undoubtedly *influences* Level 3 processes. For example, if a person is unsure of their position and their companion tells them, then they become self-localized, which we assume is accompanied by the establishment of the appropriate firing of (Level 3) place cells. People can construct cognitive maps of an environment purely based on verbal instructions [107] and generate grid-cell firing patterns based on verbally instructed imagination [108], indicating top-down influence across the levels.

## 3. Conclusion

How can the navigational toolbox framework aid research on spatial behaviour? The toolbox contains a hierarchically organized set of competences (tools) that are used to build representations that guide spatial behaviour. These are grouped in what might be considered semantic categories, pertaining to spatial information content independently of any biological substrate. In describing and hierarchically classifying the core elements of spatial representation and behaviour, the toolbox allows researchers to consider interactions between different elements of spatial processing, often at different levels in the hierarchy. Given that the toolbox provides a common frame for all species, it sheds light on the evolution of navigation and mechanisms that support navigation. Looking widely can generate new insights, a recent example being the ubiquitous role of oscillations in orientation and navigation [109,110].

One useful function of the toolbox is to provide a common functional language for neural mechanisms undergirding navigation, most closely examined in rodents and in insects. The study of neurons allows us to interrogate the inner structure of cognitive representations and to discover the modular organization that is present in these diverse taxa, and which is likely universal. By manipulating these signals experimentally, we can then determine how they are combined in order to generate complex behaviours.

The toolbox framework invites us to examine when and how the progression across levels marched in evolutionary history. By teaming the toolbox framework with a broad comparative perspective including genomic analyses, we can start to answer questions about the origins of the marvellous navigational abilities of spectacular study cases as widely disparate as bacteria, desert ants, honeybees, migratory and non-migratory birds, sea turtles, rodents and primates. An evolutionary progression up the levels is inferred, because each level is constructed with materials from the next-lower level. Level 1 may be the most ancient evolutionarily. Some later-evolved neuronal populations and circuits may work at Level 2, while a bigger conglomeration combines to form Level 3 constructs. Level 4, found almost exclusively in humans, likely evolved most recently, beginning with the appearance of symbolic thinking, linguistic communication and tool-making in early hominins, and continuing in cultural evolution at an ever-increasing pace. Level 4 devices arise quickly: GPS-based route instructions, for example, arose this century. The use of external props for navigation showcases one way in which symbol use drives cultural evolution, constituting what Jablonka & Lamb [111] have called a symbolic dimension of evolution, one aspect of a view of extended evolution [112].

The toolbox framework is incomplete in that it does not address how any form of spatial representation is put into action, which is a key challenge for movement and spatial biologists. In an age in which discussion of embodied, extended and enactive cognition is ongoing [113–116], consideration of how any spatial representation is put into action is paramount in the study of orientation and navigation. We hope that the toolbox provides a useful common framework with which to explore these issues in diverse taxa, and serves to unite disparate research frontiers on the study of wayfinding.

## Data Availability

This article has no additional data.
